# Prevalence of Depressive Symptoms in Patients With Psoriatic Arthritis: Have Numbers Changed During the COVID-19 Pandemic?

**DOI:** 10.3389/fmed.2021.748262

**Published:** 2021-11-01

**Authors:** Matthias Englbrecht, Peter Bartz-Bazzanella, Cay von der Decken, Georg Gauler, Patrick Wurth, Peer Aries, Kirsten Karberg, Christoph Kuhn, Florian Schuch, Susanna Späthling-Mestekemper, Wolfgang Vorbrüggen, Jörg Wendler, Martin Welcker, Stefan Kleinert

**Affiliations:** ^1^Freelance Data Scientist, Eckental, Germany; ^2^Medizinisches Versorgungszentrum Stolberg, Stolberg, Germany; ^3^Klinik für Internistische Rheumatologie, Rhein-Maas-Klinikum, Würselen, Germany; ^4^Rheumatologische Schwerpunktpraxis, Osnabrück, Germany; ^5^Rheumatologie im Struenseehaus, Hamburg, Germany; ^6^Praxis für Rheumatologie und Innere Medizin, Berlin, Germany; ^7^Praxis für Rheumatologie, Karlsruhe, Germany; ^8^Praxisgemeinschaft Rheumatologie-Nephrologie, Erlangen, Germany; ^9^Rheumatologist in Group Practice, Munich, Germany; ^10^Verein zur Förderung der Rheumatologie eV, Würselen, Germany; ^11^Medizinisches Versorgungszentrum für Rheumatologie Dr M Welcker GmbH, Planegg, Germany; ^12^Medizinische Klinik 3, Rheumatology/Immunology, Universitätsklinik Würzburg, Würzburg, Germany

**Keywords:** arthritis, psoriatic arthritis, depressive symptoms, COVID-19, SARS-CoV-2, depression

## Abstract

This longitudinal analysis compares the prevalence of depressive symptoms in patients with psoriatic arthritis in the context of the COVID-19 pandemic. Data from a national patient register in Germany were analyzed regarding the Patient Health Questionnaire 2 (PHQ-2) to identify cases suspicious for depression at two time points, i.e., before and during the COVID-19 pandemic. Only patients with complete concurrent information on the Disease Activity in Psoriatic Arthritis Score (DAPSA) were included in the analysis. The frequency of depressive symptoms in psoriatic arthritis patients during the COVID-19 pandemic did not differ from the prevalence rates measured before. In addition, prevalence rates for depressive symptoms did not differ when stratifying the patient sample for DAPSA levels of disease activity measured before the pandemic. These results were confirmed further in a sensitivity analysis, limiting the second PHQ-2 assessment to lockdown periods only. However, longitudinal data on the prevalence of depressive symptoms in patients with rheumatic diseases, in general, and psoriatic arthritis, in particular, are scarce in the context of the COVID-19 pandemic. For a sensible comparison of prevalence rates for depressive symptoms in the future, underlying SARS-CoV-2 infection rates and resulting local healthcare disruptions need to be taken into account, besides the potential use of different depression screening tools to evaluate resulting numbers sensibly and draw corresponding conclusions for patient care.

## Introduction

Depression is acknowledged as frequent comorbidity in inflammatory arthritis (1–4). Psoriatic arthritis (PsA) is one of the diseases summarized under the “inflammatory arthritis” label, with reported prevalence rates for depression of about 13–20% (1, 2). PsA is found in 0.1–1% of the general population and is particularly frequent in patients with psoriasis (~20%) involving the skin, nails, joints, and entheses (5). PsA patients typically face a combination of dermal and musculoskeletal symptoms impacting the health-related quality of life and social life, resulting in everyday minor and major challenges. Accordingly, recommendations for rheumatologists and dermatologists to screen and manage PsA patients regarding the underlying risk of depression were developed (6). However, regular depression screening has not been implemented into routine rheumatology care yet to help identify patients needing professional mental healthcare support. While depression screening still needed broader implementation into rheumatology care, another challenge occurred in December 2019 caused by a new coronavirus strain (SARS-CoV-2), later referred to as COVID-19. With its global spread and despite the successful initial containment of the first SARS-CoV-2 cases in late January 2020, the first wave eventually hit Germany in early March 2020, resulting in a first national shutdown by March 22nd. The temporal unavailability of face masks further stressed the situation for healthcare professionals and patients in Germany. Although resident rheumatologists and hospitals had taken quick action to refine sanitation and hygiene protocols to reduce SARS-CoV-2 infection risk for staff and patients as far as possible, many routine consultations had to be canceled and postponed. Similar and even more severe disruptive changes in rheumatology care were reported among patients with rheumatic and musculoskeletal diseases in the United States and other European countries (7, 8). In a corresponding qualitative analysis of reported perceptions given by patients referring to the pandemic, the following key themes were identified: emotions in response to the pandemic, perceptions of risks from immunosuppressive medications, protective measures to reduce risk of SARS-CoV-2 infection, and disruptions in accessing rheumatic disease medications (7). Given underlying health concerns, PsA patients perceived SARS-CoV-2 as a larger threat to their health than patients with psoriasis, whereas patients on biologics were more concerned about SARS-CoV-2 and potential outcomes of a SARS-CoV-2 infection (9). Importantly, if health concerns remain unaddressed over a longer time, disregarded feelings of helplessness may lead to depression. Systematic reviews and meta-analyses have consequently reported a high prevalence of depression in the general population, in (front-line) healthcare professionals, and patients diagnosed with SARS-CoV-2 during the pandemic (10–14). However, longitudinal data on this topic are scarce and, thus, little is known about whether the prevalence of depressive symptoms in PsA patients has increased during the COVID-19 pandemic. This retrospective analysis of PsA patient data aims to add some information to this gap and addresses whether depressive symptoms were more frequent during the COVID-19 pandemic than they were before.

## Methods

### Patient Sample and Setting

Routine clinical data from patients with an established diagnosis of PsA coming from eight centers in Germany participating in the RheumaDatenRhePort (RHADAR) register were included. RHADAR is a real-world longitudinal register for adult patients with rheumatic diseases in Germany. After informed consent, patients' pseudonymized data are added to the database. For this report, patients having consented until March 31st, 2021, were part of the data analysis. Further details on the RHADAR register can be found elsewhere (15). Symptoms of depression were assessed using the Patient Health Questionnaire-2 (PHQ-2), a brief two-item depression screening tool, which had previously been validated in patients with rheumatoid arthritis, demonstrating good sensitivity and specificity (16, 17). The PHQ-2 sum score ranges from 0 (best) to 6 (worst), with scores ≥ 3 indicating depressive symptomatology. The RHADAR database was queried for patients who had had a first PHQ-2 assessment in the 12 months preceding the confirmation of the first SARS-CoV-2 cases in Germany (T1: January to December 2019, first SARS-CoV-2 cases in Germany: January 27th 2020) and had another assessment during the pandemic, i.e., after the first national lockdown taking effect on March 22nd 2020 (T2). If multiple PHQ-2 assessments for T1 were available, the first of them was chosen. The second assessment was supposed to be including PsA patients whose appointments had previously been postponed to cover changes in affective mood, resulting from potential healthcare disrupting effects. In addition, all patients included had to have an assessment of the Disease Activity in Psoriatic Arthritis Score (DAPSA) corresponding to each PHQ-2 assessment (18). Additional sample characteristics include information on sex, age, disease duration, the Hannover Functional Ability Questionnaire (HFAQ) as a measure of physical functioning, which is equivalent to the Health Assessment Questionnaire Disability Index (HAQ-DI), the Body Surface Area (BSA) for skin involvement, and anti-rheumatic treatment, aggregated by drug class (19). Besides the RHADAR inclusion criteria and the mandatory availability of data on PHQ-2 and DAPSA before and during the SARS-CoV-2 pandemic, no further inclusion or exclusion criteria were applied.

### Statistical Data Analysis and Analysis Software

Descriptive characteristics of quantitative variables are presented as mean ± standard deviation and as absolute frequencies (per cent) for nominal data if not stated otherwise. Data on prescribed medication were aggregated into the following drug classes: conventional-, targeted synthetic-, and biological disease-modifying anti-rheumatic drugs (cDMARDs, tsDMARDs, bDMARDs), non-steroidal anti-inflammatory drugs (NSAIDs), and glucocorticoids (GCs). Due to combination therapies, the reported total number of prescriptions may exceed the sample size. Missing values were not imputed to preserve the original information of the available raw data. Differences in the prevalence of symptoms of depression and the prescription frequencies of aggregated standard anti-rheumatic therapies for PsA were investigated by McNemar's tests for paired nominal data, including Yate's correction for continuity. PHQ-2 sum scores were dichotomized using a sum score cutoff ≥ 3, which is the standard threshold to identify cases suspicious for depression. For both time points, before and during the pandemic, the frequencies of the dichotomized PHQ-2 scores were compared. In a subsequent step, this analysis was repeated, stratified for DAPSA levels of disease activity, i.e., ≤ 4 for remission, >4 and ≤14 for low disease activity, >14 and ≤ 28 for moderate disease activity and >28 for high disease activity (20). The stratified PHQ-2 analysis as well as the analysis of drug class-specific prescription frequencies were adjusted for multiple comparisons using Bonferroni-Holm correction to control type I error probability. Inferential test results include test coefficient, *p*-value and effect size, i.e., odds ratios with corresponding 95% confidence intervals. McNemar test-related odds ratios were calculated from the division of the off-diagonal cells in the respective contingency table (Supplementary File). An additional sensitivity analysis regarding PHQ-2-related outcomes was conducted to investigate whether the choice of the T2 assessment impacted the results. For this analysis, only patients with a second PHQ-2 assessment during lockdown periods were included. The data analysis was conducted using R (version 4.1.0.) and RStudio IDE (version 1.4.1103) (21, 22). *P* ≤ 0.05 were considered statistically significant in cases with no multiple testing adjustment.

## Results

### Description of the Patient Sample

Eighty-nine PsA patients with 48 female patients (53.93%) and 41 male patients (46.07%) were included in the analysis. On average, patients were 54.16 ± 11.66 years of age and had a disease duration of 9.33 ± 9.02 years at T1. Mean T1 DAPSA was 10.38 ± 14.56, suggesting patients had low disease activity on average, whereas the subgroup analysis revealed the following distribution across DAPSA categories: 38 (42.70%) remission, 31 (34.83%) low disease activity, 12 (13.48%) moderate disease activity, and 8 (8.99%) high disease activity. Mean DAPSA at T2 was 7.91 ± 10.31, whereas DAPSA confidence intervals for both time points indicated comparable disease activity (Table 1). With potential scores ranging from 0 (worst) to 100 (best), average HFAQ-scores showed mild impairment of physical functioning at both time points (T1: 80.83 ± 21.77, T2: 82.51 ± 20.30). Further descriptive information is presented in Table 1.

**Table 1:** Descriptive sample characteristics before and during the SARS-CoV-2 pandemic.

	***N* (valid)**	**% (valid)**	***N* (NA)**	**% (NA)**	**Mean**	**Lower bound 95%CI**	**Upper bound 95%CI**	**SD**
**T1**								
Age	89	100.00	0	0.00	54.16	51.74	56.58	11.66
Disease duration (years)	89	100.00	0	0.00	9.33	7.45	11.20	9.02
DAPSA	89	100.00	0	0.00	10.38	7.36	13.41	14.56
TJC (DAPSA)	89	100.00	0	0.00	3.03	1.29	4.77	8.38
SJC (DAPSA)	89	100.00	0	0.00	1.24	0.39	2.08	4.05
NRS Disease Activity (DAPSA)	89	100.00	0	0.00	2.84	2.27	3.41	2.75
NRS Pain (DAPSA)	89	100.00	0	0.00	2.63	2.09	3.17	2.61
CRP	89	100.00	0	0.00	0.64	0.36	0.92	1.35
BSA	29	32.58	60	67.42	0.45	0.12	0.78	0.91
HFAQ	86	96.63	3	3.37	80.83	76.22	85.43	21.77
**T2**								
Age	89	100.00	0	0.00	55.27	52.85	57.69	11.63
Disease duration	89	100.00	0	0.00	10.44	8.56	12.32	9.06
DAPSA	89	100.00	0	0.00	7.91	5.77	10.05	10.31
TJC (DAPSA)	89	100.00	0	0.00	1.96	0.56	3.35	6.70
SJC (DAPSA)	89	100.00	0	0.00	0.76	0.29	1.24	2.29
NRS Disease Activity (DAPSA)	89	100.00	0	0.00	2.35	1.90	2.79	2.14
NRS Pain (DAPSA)	89	100.00	0	0.00	2.38	1.90	2.87	2.34
CRP	89	100.00	0	0.00	0.46	0.34	0.58	0.58
BSA	16	17.98	73	82.02	0.75	0.29	1.21	0.93
HFAQ	86	96.63	3	3.37	82.51	78.22	86.80	20.30

*CI, confidence interval; SD, standard deviation; DAPSA, Disease Activity Psoriatic Arthritis Score; TJC, Tender Joint Count (68 joints); SJC, swollen joint count (66 joints); NRS, Numerical Rating Scale; ESR, erythrocyte sedimentation rate; CRP, c-reactive protein; BSA, Body Surface Area; HFAQ, Hannover Functional Ability Questionnaire. T1, January—December 2019; T2, March 22nd 2020–March 31st 2021*.

At T1, cDMARDs were the most frequent drug (*n* = 57, 64.04%), followed by NSAIDs (*n* = 45, 50.56%), bDMARDs (*n* = 35, 39.33%), GCs (*n* = 15, 16.85%), and tsDMARDs (*n* = 1, 1.12%). Complete data on anti-rheumatic medication was available for 88 (98.88%) patients at T1 and 85 (95.51%) patients at T2. Except for GCs [χ^2^_(1)_ = 7.11, *p* = 0.008], which were prescribed less frequent at T2 than at T1, prescription frequencies between the time points of interest did not differ (see section 1 of Supplementary File for further information). The corresponding odds ratio for GCs could not be calculated as division by 0, given by the contingency table, is undefined. However, the change in the prescription frequency of GCs remained significant after multiple testing adjustments (critical p_adj_ = 0.01). Importantly, our results did indicate that neither cDMARDS [χ^2^_(1)_ = 0.36, *p* = 0.55, 95%CI_OR_ = 0.17–1.95] nor bDMARDs [χ^2^_(1)_ = 0, *p* = 1.0, 95%CI_OR_ = 0.14–7.10] were prescribed more or less often during the pandemic than before.

### Prevalence of Depressive Symptoms

PHQ-2 frequency analysis showed that the majority of the patients in our sample had a PHQ-2 sum score ≤ 2, which is below the cutpoint for an indication of depressive symptoms at both time points (T1: *n* = 74, 83.15%; T2: *n* = 76, 85.39%). A total of 15 (T1) and 13 (T2) patients were found to show depressive symptomatology. Accordingly, the prevalence of symptoms of depression, identified by a PHQ-2 sum score ≥ 3, was 16.85% at T1, and 14.61% at T2, respectively. The corresponding inferential McNemar analysis did not reveal any significant changes regarding depressive symptoms when comparing data from before SARS-CoV-2 to data during the pandemic [χ^2^_(1)_ = 0.06, *p* = 0.803, 95%CI_OR_ = 0.48–3.45]. Corresponding frequency data even suggested a slight (although non-significant) decrease in prevalence rates. When PHQ-2 data were stratified for DAPSA levels of disease activity, each of the resulting four categories did not indicate any significant changes in prevalence rates for depressive symptoms. With *p*-values ranging from 0.617 to 1.000 and 95% odds ratio confidence intervals encompassing 1, prevalence rates seemed equal over time irrespective of the DAPSA stratification for disease activity. Further details on PHQ-2 test results are shown in Tables 2, 3; contingency tables are shown in section 2 of the Supplementary File. The sensitivity analysis reduced longitudinal comparisons only to those patients that had their second assessment during one of the two national lockdowns. The corresponding results confirmed the previous findings, again, suggesting prevalence rates for depressive symptoms before and during the SARS-CoV-2 pandemic to be comparable in our sample [χ^2^_(1)_ = 0.25, *p* = 0.617, 95%CI_OR_ = 0.31–28.84; χ^2^_(1)_ = 1.50, *p* = 0.221, 95%CI_OR_ = 0.58–42.80; see section 3 of the Supplementary File for corresponding contingency tables].

**Table 2:** Frequency distribution of PHQ-2 sum scores before and during the SARS-CoV-2 pandemic (n_total_ = 89).

	**T1**			**T2**		
**PHQ-2**	***n***	**%**	**% (cumulative)**	***n***	**%**	**% (cumulative)**
0	39	43.82	43.82	41	46.07	46.07
1	12	13.48	57.30	11	12.36	58.43
2	23	25.84	83.15	24	26.97	85.39
3	4	4.49	87.64	3	3.37	88.76
4	6	6.74	94.38	6	6.74	95.51
5	1	1.12	95.51	2	2.25	97.75
6	4	4.49	100.00	2	2.25	100.00
**≥ 3**	**15**	**16.85**	**–**	**13**	**14.61**	

*T1, January–December 2019; T2, March 22nd 2020–March 31st 2021. Bold values represent column sums for n and % in the rows with a PHQ-2 score of 3 or higher, i.e., PHQ-2 scores of 3, 4, 5, and 6*.

**Table 3:** McNemar tests for dichotomized PHQ-2 scores for the total sample and stratified by initial DAPSA levels at the first assessment before and during the SARS-CoV-2 pandemic.

**Matched sample comparison**	***n***	**χ^**2**^**	**df**	***p*-value**	**OR**	**Lower bound 95%CI**	**Upper bound 95%CI**
Total sample	89	0.06	1	0.803	1.286	0.479	3.452
DAPSA—REM (T1)	38	0.25	1	0.617	0.333	0.035	3.205
DAPSA—LDA (T1)	31	0.00	1	1.000	1.000	0.141	7.099
DAPSA—MDA (T1)	12	0.25	1	0.617	3.000	0.312	28.842
DAPSA—HDA (T1)	8	0.25	1	0.617	3.000	0.312	28.842

*df, degrees of freedom; OR, odds ratio (effect size); CI, confidence interval; DAPSA, Disease Activity Psoriatic Arthritis Score; REM, Remission (DAPSA ≤ 4); LDA, low disease activity (DAPSA > 4 and ≤ 14); MDA, moderate disease activity (DAPSA >14 and ≤ 28); HDA, high disease activity (DAPSA > 28)*.

## Discussion

Regarding the prevalence of depressive symptoms identified by the PHQ-2, our results align with the numbers given by recent systematic reviews in PsA (1, 2). Surprisingly, the results of our data analysis suggest depressive symptoms not to occur more often during the SARS-CoV-2 pandemic compared to the 2019 data for patients having completed a depression screening at both time points. Except for GCs, that were prescribed less frequent at T2, our findings showed that anti-rheumatic medication remained unchanged in the vast majority of our sample. Thus, switches of anti-rheumatic medication do not seem to be a reason for the stable prevalence rates. With the help of a sensitivity analysis regarding the choice of the T2 time point, we were also able to show that results remained unchanged when limiting the second assessment to lockdown periods only. However, given the challenges the healthcare system and rheumatologists were facing, particularly during the first national lockdown, the corresponding sample sizes were smaller (T2: first lockdown: *n* = 18, T2: second lockdown: *n* = 50). A post-lockdown study confirmed a similar prevalence in patients with inflammatory arthritis and gave a plausible explanation of why depressive symptoms might not occur more frequently during the SARS-CoV-2 pandemic (23). According to Ciaffi et al., patients with inflammatory arthritis showed higher resilience scores than study participants from the general population—independent of the patients' age or disease duration (23). Resilience is a psychological construct implying resistance to environmental risk experiences or overcoming stress or adversity (24). The authors of the study above hypothesize whether higher resilience might result from the necessity for patients to adapt to everyday hassle resulting from inflammatory arthritis, in turn, training their abilities to cope more effectively in challenging situations than individuals in the general population (23). Adding to these findings, another study in the U.S. general population revealed that getting outside more often, exercising more often, sleeping better, praying more often, and perceiving social support from friends, family, and significant others were also related to higher resilience (25). Though we did not measure these characteristics, an additional positive effect of these factors seems conceivable in PsA patients, too. However, in contrast to our findings, a multi-national European study including 1,800 patients with rheumatic and musculoskeletal diseases reported a proportion of 45.9% of the participants at risk of depression during the COVID-19 pandemic (8). These results suggest that prevalence rates of depressive symptoms could be more than double the numbers according to our findings. Several reasons may serve as conceivable explanation of this difference: Firstly, the study mentioned above had a different sample composition, including only 9.1% of PsA patients and were given a different depression screening questionnaire returning two outcome categories for depressive symptoms, which were conflated for the analysis (borderline cases and cases suspicious for depression). The PHQ-2 in our analysis returns a single category for depressive symptoms and has no borderline category. These factors make a direct comparison of results difficult and can lead to considerably different prevalence rates. Secondly, the national healthcare systems across Europe are structured differently and were stressed to an individually different degree during the COVID-19 pandemic, depending on the local rate of infection on the one hand and available healthcare resources on the other. The study by Garrido-Cumbrera et al. included participants from several countries such as Spain, Portugal, France, Italy, or the UK which all had peak 7-day case rates per 100.000 that were twice as high as the numbers in Germany, at least (26). Corresponding limited healthcare resources including considerable disruptions having an impact on the availability of necessary (pain-related) medication and patient care are another feasible explanation for the higher prevalence rates of depressive symptoms found in these countries. The implications from this comparison are important to understand the generalizability of the results coming from our analysis. A mere cross-country comparison of numbers reflecting the occurrence of depressive symptoms in the context of the COVID-19 pandemic does not match the complexity of the issue. Data on prevalence rates of depressive symptoms during COVID-19 need to be interpreted in the context of the impact the pandemic had on the availability of healthcare resources in each region or country of interest. For our analysis, we have chosen longitudinal PHQ-2 data from PsA patients including DAPSA as common measure of disease activity in PsA. However, information on psoriatic skin involvement by BSA were only available for a less than a third of the total patient sample. This might reflect rheumatologists' reluctance for an exact skin documentation, limiting comparability to other PsA samples investigating a similar research question to the musculoskeletal component and the more general visual analog scale for patient-reported global assessment. Furthermore, with including 89 patients with sufficient PHQ-2 and DAPSA documentation, our sample might not be generalizable to other countries or patient samples. Baseline PHQ-2 values preceding the T1 assessment were not included in the manuscript given that we felt that a period of 12 months preceding the COVID-19 pandemic for definition of the T1 assessment was sufficient. Regarding DAPSA, the patients in our sample seemed to have milder disease than the norming sample that was used to define DAPSA levels of disease activity by a quartile split (20). This finding may result from the majority of centers contributing data to the RHADAR register being resident rheumatologists adding patients with lower disease activity to the database than hospitals potentially would. However, since we were able to demonstrate, that the prevalence of depressive symptoms remained similar across DAPSA categories and is independent from the choice of timing for the second assessment as well, we consider our results reliable given similar numbers from previous large-scale systematic reviews. Given comparable results regarding 95% confidence intervals of mean PHQ-2 scores of database patients with a single PHQ-2 assessment at T1 (i.e., patients who were not included into the analysis) and the patient sample presented, we assume that patients with a single PHQ-2 assessment at T1 did not show higher depressiveness on average than the patients included into the analysis of this manuscript (95%CI only T1: 1.44–1.68, 95%CI patient sample: 1.04–1.72). Hence, a potential selection bias resulting from a mandatory T1 and T2 documentation as inclusion criterion is unlikely. Unfortunately, longitudinal data for comparing the prevalence of depressive symptoms before and during SARS-CoV-2 are scarce in rheumatology, yet and, thus, would merit further, preferably large-scale data input leading to improved insights of what kind of circumstances still can be handled by improved resilience capabilities of patients with rheumatic disorders and at which point even these might not suffice to bolster challenges such as those resulting from the pandemic.

In conclusion, our analysis showed similar prevalence rates of depressive symptoms before and during SARS-CoV-2 in PsA patients in Germany, irrespective of the patients' DAPSA category or the timing of the during COVID-19 assessment. To the best of our knowledge, this is the first longitudinal investigation of depressive symptoms in patients with PsA during the COVID-19 pandemic.

## Data Availability Statement

The data analyzed in this study is subject to the following licenses/restrictions: The raw data for this analysis are not openly available given the patients' consent, allowing for the publication of aggregated data only. However, information on aggregated raw data is given in the contingency tables of the Supplementary Material. Requests to access these datasets should be directed to pmanagement@statscoach.de.

## Ethics Statement

Ethical review and approval was not required for the study on human participants in accordance with the local legislation and institutional requirements. The patients/participants provided their written informed consent to participate in this study.

## Author Contributions

All authors listed have made a substantial, direct and intellectual contribution to the work, and approved it for publication.

## Funding

This study was funded by the RHADAR GbR (A Network of Rheumatologists), Bahnhofstr. 32, 82152 Planegg, Germany. RHADAR GbR received honoraria from UCB Pharma GmbH, Sandoz Deutschland/Hexal AG, Lilly GmbH, and Galapagos Biopharma Germany GmbH, and research support from Novartis Pharma GmbH. The funders were not involved in the study design, collection, analysis, interpretation of data, the writing of this article or the decision to submit it for publication.

## Conflict of Interest

WV, CD, SK, PB-B, CK, KK, SS-M, PA, and MW are members of RheumaDatenRhePort GbR. RHADAR GbR received honoraria from UCB Pharma GmbH, Sandoz Deutschland/Hexal AG, Lilly GmbH, and Galapagos Biopharma Germany GmbH, and research support from Novartis Pharma GmbH. ME received remunerations from RheumaDatenRhePort GbR for statistical data analyses and consultation for previous projects. The remaining authors declare that the research was conducted in the absence of any commercial or financial relationships that could be construed as a potential conflict of interest.

## Publisher's Note

All claims expressed in this article are solely those of the authors and do not necessarily represent those of their affiliated organizations, or those of the publisher, the editors and the reviewers. Any product that may be evaluated in this article, or claim that may be made by its manufacturer, is not guaranteed or endorsed by the publisher.
