# Supplementary material for: Prevalence of Depressive Symptoms in Patients With Psoriatic Arthritis: Have Numbers Changed During the COVID-19 Pandemic?
Source: Front Med (Lausanne). 2021 Nov 1;8:748262. doi: 10.3389/fmed.2021.748262 (PMC8591045; doi:10.3389/fmed.2021.748262)
Supplement: Supplementary file 1 [file Data_Sheet_1.DOCX]

**Supplementary Material**

**Section 1. 2 x 2 contingency tables for McNemar tests regarding changes in prescription of anti-rheumatic medication aggregated in drug classes**

(T1: before SARS-CoV-2 in Germany in 2019, T2: After March 22^nd^ 2020 corresponding to the beginning of the first lockdown in Germany)

**cDMARDs (n = 84)**

|  | T2: prescribed | T2: not prescribed |
| --- | --- | --- |
| T1: prescribed | 51 | 4 |
| T1: not prescribed | 7 | 22 |

**tsDMARDs (n = 84)**

|  | T2: prescribed | T2: not prescribed |
| --- | --- | --- |
| T1: prescribed | 1 | 0 |
| T1: not prescribed | 0 | 83 |

**bDMARDs (n = 84)**

|  | T2: prescribed | T2: not prescribed |
| --- | --- | --- |
| T1: prescribed | 32 | 2 |
| T1: not prescribed | 2 | 48 |

**NSAIDs (n = 84)**

|  | T2: prescribed | T2: not prescribed |
| --- | --- | --- |
| T1: prescribed | 40 | 3 |
| T1: not prescribed | 0 | 41 |

**GCs (n = 84)**

|  | T2: prescribed | T2: not prescribed |
| --- | --- | --- |
| T1: prescribed | 4 | 9 |
| T1: not prescribed | 0 | 71 |

**McNemar tests for differences in prescription frequencies between T1 and T2**

| **Drug class** | **n** | **χ^2^** | **df** | **p-value** | **OR** | **Lower bound  95%CI** | **Upper  bound  95%CI** |
| --- | --- | --- | --- | --- | --- | --- | --- |
| cDMARDs | 84 | 0.364 | 1 | 0.546 | 0.571 | 0.167 | 1.952 |
| tsDMARDs | 84 | Na | 1 | Na | Na | Na | Na |
| bDMARDs | 84 | 0.000 | 1 | 1.000 | 1.000 | 0.141 | 7.099 |
| NSAIDs | 84 | 1.333 | 1 | 0.248 | Inf | Na | Inf |
| GC | 84 | 7.111 | 1 | 0.008 | Inf | Na | Inf |

**Section 2. 2 x 2 contingency tables for McNemar tests shown in Table 3**

Dichotomized PHQ-2 scores indicating depressive symptomatology (PHQ-2 ≥ 3)
(T1: before SARS-CoV-2 in Germany in 2019, T2: After March 22^nd^ 2020 corresponding to the beginning of the first lockdown in Germany)

**Total sample (n = 89)**

|  | T2: PHQ-2 ≥ 3 | T2: PHQ-2 < 3 |
| --- | --- | --- |
| T1: PHQ-2 ≥ 3 | 6 | 9 |
| T1: PHQ-2 < 3 | 7 | 67 |

**DAPSA – Remission (n = 38)**

|  | T2: PHQ-2 ≥ 3 | T2: PHQ-2 < 3 |
| --- | --- | --- |
| T1: PHQ-2 ≥ 3 | 1 | 1 |
| T1: PHQ-2 < 3 | 3 | 33 |

**DAPSA – low disease activity (n = 31)**

|  | T2: PHQ-2 ≥ 3 | T2: PHQ-2 < 3 |
| --- | --- | --- |
| T1: PHQ-2 ≥ 3 | 3 | 2 |
| T1: PHQ-2 < 3 | 2 | 24 |

**DAPSA – moderate disease activity (n = 12)**

|  | T2: PHQ-2 ≥ 3 | T2: PHQ-2 < 3 |
| --- | --- | --- |
| T1: PHQ-2 ≥ 3 | 1 | 3 |
| T1: PHQ-2 < 3 | 1 | 7 |

**DAPSA – high disease activity (n = 8)**

|  | T2: PHQ-2 ≥ 3 | T2: PHQ-2 < 3 |
| --- | --- | --- |
| T1: PHQ-2 ≥ 3 | 1 | 3 |
| T1: PHQ-2 < 3 | 1 | 3 |

**Section 3. 2 x 2 contingency tables for McNemar tests used for sensitivity analysis regarding timing for second assessment – T2 limited to assessments during national lockdowns** **only**

(T1: before SARS-CoV-2 in Germany in 2019, T2: Lockdown period only)

**T2: First lockdown (March 22^nd^ 2020 – May 4^th^ 2020)**

|  | T2: PHQ-2 ≥ 3 | T2: PHQ-2 < 3 |
| --- | --- | --- |
| T1: PHQ-2 ≥ 3 | 2 | 3 |
| T1: PHQ-2 < 3 | 1 | 12 |

**T2: Second lockdown (December 16^th^ 2020* – March 31^st^ 2021**)**

|  | T2: PHQ-2 ≥ 3 | T2: PHQ-2 < 3 |
| --- | --- | --- |
| T1: PHQ-2 ≥ 3 | 5 | 5 |
| T1: PHQ-2 < 3 | 1 | 39 |

* On November 2^nd^ 2020, a so-called “lockdown light” was initially issued in Germany. However, the SARS-CoV-2 incidence rate further increased leading to additional containment and distancing regulations that were announced on Sunday, December 13^th^ 2020 and taking effect from December 16^th^ 2020 onwards. These conditions were stricter and were comparable to the regulations during the first national lockdown.

**Date of RHADAR spring database lock was before the end of the restrictions of the second national lockdown which is why the date of the database lock was chosen.
